# The effects of pronated foot posture and medial heel and forefoot wedge orthoses on static balance in older people

**DOI:** 10.1186/1757-1146-7-S1-A17

**Published:** 2014-04-08

**Authors:** Fateme Hemmati, Saeed Forghany, Christopher Nester

**Affiliations:** 1 Musculoskeletal Research Centre, Isfahan University of Medical Sciences, Iran; 2 Centre for Health Sciences Research, University of Salford, UK

## Background

Aging has been associated with increasing foot pronation [1] and changes in foot mobility and posture which may influence standing balance [2,3]. Orthotic interventions change foot posture [4] and load distribution under the foot [5] and therefore may have important effects on balance in older people.

## Objective

To investigate whether a pronated foot posture is associated with poorer standing balance in older people and whether medial heel and forefoot wedge orthoses affect their standing balance.

## Design

Between groups, repeated-measures design.

## Methods

Ten healthy older people with a pronated foot posture (age 67.1± 5.5 years) and sixteen healthy elderly with normal foot posture (age 67.1± 5.9 years) were recruited.The Foot Posture Index (FPI) was used to determine pronated and normal foot posture. Static balance in double limb stance was assessed using Kistler force plate measures during four shod conditions: 1) 5° medial heel and forefoot wedge (W5); 2) 8° medial heel and forefoot wedge (W8); 3) Control insole for W5 (flat EVA base with the same thickness as W5 (NW5)); 4) Control insole for W8 (flat EVA base with the same thickness as W8 (NW8)). Each of the four cases was completed with eyes open and eyes closed. The center-of-pressure (COP) total excursion and mean velocity and area of 95% confidence ellipse were derived as measures of standing balance.

## Results

**Figure 1:**
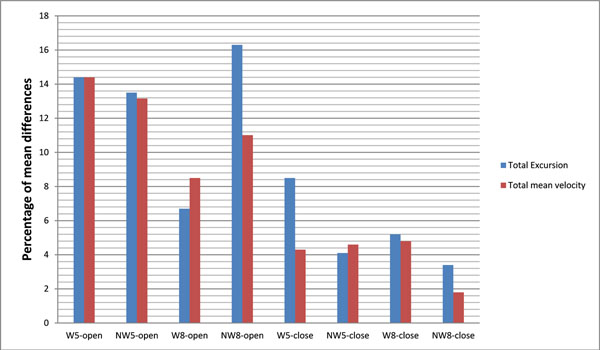
Percentage of mean differences in COP parameters between participants with a pronated foot type and those with normal foot type across different orthoses and eyes conditions (open and close)

Participants with a pronated foot type (Mean FPI: 7.5) demonstrated greater total excursion (298.19±28.59mm versus 262.69±22.92mm) and total mean velocity (11.78±1.41mm.s^-1^ versus 10.41±1.13mm.s^-1^), and larger ellipse area (630.81±244.19mm^2^ versus 298.15±195.79 mm^2^), compared with participants with normal foot type (Mean FPI: 3.8) during normal standing, but this did not reach statistical significance (p>0.05) (Figure 1). There was a significant main effect for eyes open (p<0.05) with the total excursion (290.0 ± 14.7mm versus 321.9 ± 14.1mm) and mean velocity of COP in ML (8.9 ± 0.5mm.s^-1^ versus 10.2 ± 0.6mm.s^-1^) being significantly lower.

There were no statistically significant effects from the four orthoses in the pronated nor the normal foot types (p<0.05). There were no significant differences in interaction of all conditions (foot posture × eye condition × orthoses) (p<0.05).

## Conclusion

A trend towards less stable balance was observed in pronated foot type but this was not significant. Use of orthoses had no effect on balance parameters including negating the effects of eyes closed. Orthoses showed no negative effects on standing balance and therefore do not pose a threat to balance (e.g. if they are used for another purpose).
